# Full Valley and Spin Polarizations in Strained Graphene with Rashba Spin Orbit Coupling and Magnetic Barrier

**DOI:** 10.1038/srep21590

**Published:** 2016-02-22

**Authors:** Qing-Ping Wu, Zheng-Fang Liu, Ai-Xi Chen, Xian-Bo Xiao, Zhi-Min Liu

**Affiliations:** 1 Department of Applied Physics, East China Jiaotong University, Nanchang 330013, China; 2 School of Computer Science, Jiangxi University of Traditional Chinese Medicine, Nanchang 330004, China

## Abstract

We propose a graphene-based full valley- and spin-polarization device based on strained graphene with Rashba spin orbit coupling and magnetic barrier. The underlying mechanism is the coexistence of the valley and single spin band gaps in a certain Fermi energy. By aligning the Fermi energy in the valley and single spin band gaps, remarkable valley- and spin-polarization currents can be accessed.

The realization of stable single-layer carbon crystals, called graphene, has led to an intensive investigation of electronic properties of graphene^1^,^2^. Charge carriers in a sheet of single-layer graphene behave like “relativistic” chiral massless particles with a “light speed” equal to the Fermi velocity and possess a gapless linear spectrum at the *K* and *K’* points^1^,^3^. Graphene has been regarded as a promising material for future nanoelectronic devices, particularly in the field of spintronics, as it is expected to have long spin decoherence and relaxation times^4^,^5^. By making graphene ferromagnetic through magnetic proximity effect, research^6^,^7^,^8^,^9^ has demonstrated the tunable spin transport in ferromagnetic graphene, rendering the activity of graphene in future spin-electronic applications. Recently Liu *et al*.^10^ found that the transport in a two-dimensional graphene structure modulated by the spin orbit coupling and a local strain follows high spin polarization. Graphene in addition to the spin of the electron has two more degrees of freedom: sublattice and valley pseudospin. Valley based electronics, also known as valleytronics^11^,^12^, uses the valley degree of freedom as a carrier of information similar to the way spintronics uses electron spin. It was shown recently that graphene nanoribbons with zigzag edges^11^,^13^ can be used as a valley filter. Another promising method to achieve the valley-polarized output current is to construct a bulk graphene modulated by a double barrier structure consisting of electric and vector potentials^14^. And recently research^15^ suggested that a strained graphene modulated by a finite magnetic superlattice allows a coexistence of insulating transmission gap of one valley and metallic resonant band of the other. Accordingly, a full valley-polarization current appears.

Although the single spin and valley polarizations in graphene have been extensively studied, the works, realizing simultaneously both spin and valley polarizations for graphene, are still few^16^. In this Letter, we propose a graphene-based valley/spin polarization device by placing strained graphene with Rashba spin-orbit coupling (RSOC) and magnetic barrier. Our motivation for studying such a structure is twofold. First, the strain combined with magnetic barrier in graphene can generate different transmission gap for *K* and 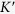 valley. Secondly, RSOC can induce spin flip and spin energy gap. Thus, the spin energy gap of one valley maybe overlap with the transmission gap of the other valley. In other words, only one spin state from one valley can be transmitted and any other spin states are forbidden, provided the Fermi energy located in the overlap region. In this work, combining the local strain, magnetic barrier and RSOC, we show that full valley- and spin-polarization currents can be accessed simultaneously, and the receiving spin polarization can be easily tuned by the strain and magnetization strength, which offers the very feature to construct spin/valley filter.

## Computational Models and Methods

**Figure 1:**
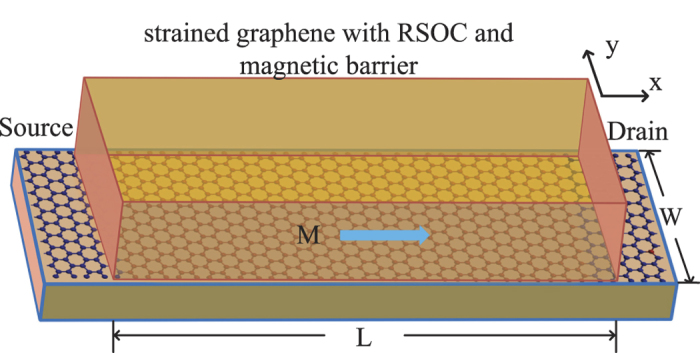
A schematic depiction of the graphene-based electronic device. The source and drain electrodes are normal graphene. The central region is stained graphene with RSOC and magnetic barrier. The magnetic field is created by a ferromagnetic metal (FM) strips with *x*-axis magnetizations (blue arrows) depositing on top of the strained graphene region. Where *W* is the width of the graphene sample in the *y* direction, L is the length of strained region.

The graphene-based system under consideration includes the strain, RSOC and magnetic barrier, as depicted in Fig. 1. The local strain can be induced by a tension along the *x* direction applied on the graphene and is homogeneous along the *y* axis. The elastic deformation can be treated as a perturbation to the hopping amplitudes and acts as a gauge potential 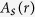
17. Explicitly, we have 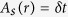, where *δt* parametrizes the strain by its effect on the nearest neighbor hopping, 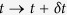 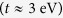. Within a tight-binding formulation of the electronic motion^12^, the change of hopping energy can be achieved 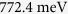, if the bond length increase 10% under strain^9^,^17^. RSOC can be induced by growing graphene on a Ni surface by catalytic methods^18^, and study shows that Au intercalation at the graphene-Ni interface creates a giant spin-orbit splitting 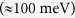 in the graphene Dirac cone up to the Fermi energy. We consider the RSOC strength 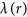 are constant in the strained region and vanishes otherwise. The magnetic field is assumed to vary only along the *x* axis, which can be generated by depositing ferromagnetic metal or superconducting materials on top of the dielectric layer, as is the way in semiconductor heterostructures^19^,^20^. The induced magnetic field can be approximately described^21^ by the vector potential 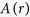. The vector potential 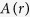 is scaled in units of 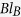, *B* is the strength of the local magnetic field, 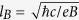
22. We assume that the sample width 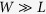, so that edge details are not important. Including RSOC and magnetic barrier together, the low-energy effective Hamiltonian for strained graphene can be written as


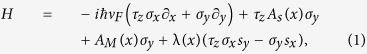


here 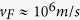 is the Fermi velocity, 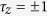 for *K*, 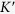 valleys, 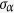 Pauli matrices act in the A, B space, and 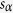 is Pauli matrices acting on the electron’s spin, and here 
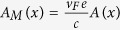
22. Upon diagonalization of the Hamiltonian, we obtain the energy dispersion for both valleys


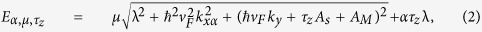


here 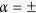 is the sign of the spin projection along the in-plane direction orthogonal to the propagation direction, 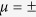 stands for the conduction (+) and valence (−) bands. 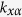 is the longitudinal wave vector, 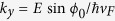, here 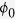 is the incident angle. For an electron in 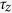 valley with energy *E* and incident angle 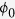, we denote the transmission probability as 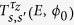, here the indexes 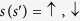 specify the incoming and transmitting spin orientations respectively. The transfer matrix method^23^ is adopted to calculate 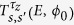. Once the transmission probability is known, one can determine the spin-dependent conductance^24^.


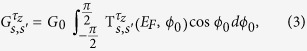


here 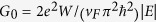, *W* is the width of the graphene sample in the *y* direction. The valley, spin resolved and total conductance are defined as


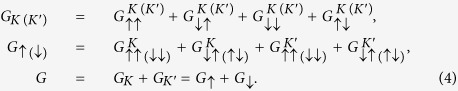


We also introduce valley and spin polarizations 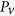 and 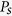



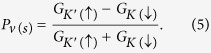


## Results and Discussion

**Figure 2:**
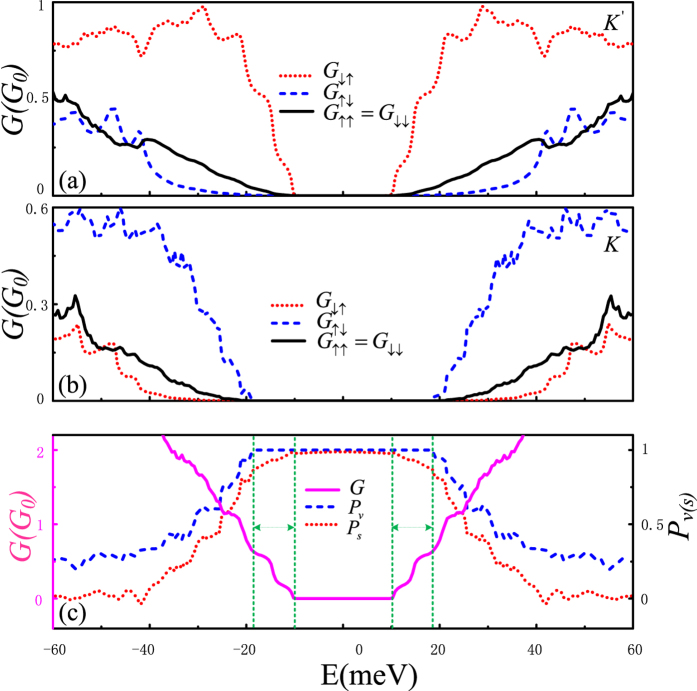
(**a**,**b**) Valley- and spin-dependent conductance in unit of 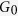, (**c**) total conductance/spin polarization/valley polarization as functions of the Fermi energy. The strengths of strain, magnetic and RSOC are: 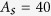
meV, 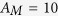 meV, 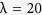 meV, and the total width 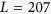 nm.

Figures 2(a,b) display spin resolved conductance for 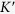 and *K* valleys as function of the Fermi energy. As the incident energy increases, for 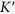 valley [Fig. 2(a)], the conductance shows an obvious blocked region in the Fermi energy regions −10.2 meV < *E*_*F*_ < 10.2 meV. For *K* valley [Fig. 2(b)], the conductance is completely suppressed in a wider energy ranges [−18.9 meV, 18.9 meV]. Figure 2(a,b) also show that two spin-conserved conductances of each valley coincide for all Fermi energy, but the two spin-flipped conductance are different because the Hamiltonian of single valley system is no longer invariant under operation 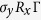
10, where Γ is time-reversal operator^25^. It is worth to note that in the energy ranges 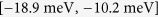 and 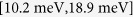, the conductance is contributed mainly by its spin- flipped component 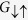 from the 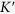 valley, while the others conductances are greatly suppressed, even the transport channels from *K* valley are forbidden wholly. Such transport property leads to that there are not only full valley polarization and high spin polarization but also obvious total conductance in the energy ranges 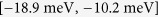 and 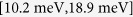 [seen in the green dashed lines in Fig. 2(c)]. In addition, the extremely high spin and valley polarizations also are present in the energy region −10.2 meV < *E*_*F*_ < 10.2 meV, but the rather small conductance makes it impossible for either detection or applications. From Fig. 2(c), one can also find that the valley and spin polarizations decrease gradually and eventually decay to zero with increasing the energy.

**Figure 3:**
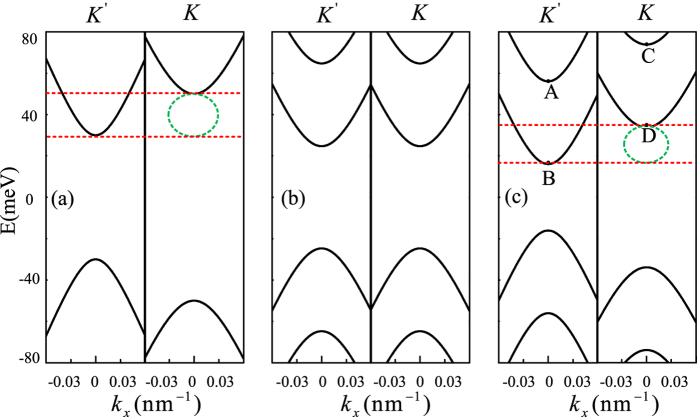
Bulk states band structure with 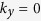, for (**a**) 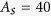 meV, 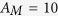 meV, 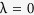 meV; (**b**) 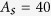 meV, 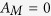 meV, 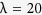 meV; (**c**) 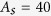 meV, 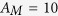 meV, 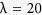 meV.

In this work, we propose and demonstrate that valley and spin filters can be simultaneously achieved in a strained graphene with RSOC and magnetic barrier. Such a remarkable result can be understood by the band structures in each region. For comparison, we first show the band structures near the 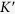 and *K* points in strained graphene with only magnetic barrier [Fig. 3(a)] and only RSOC [Fig. 3(b)]. In Fig. 3(a), a band gap is induced, and the band gap for *K* valley is wider than that of 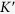 valley since the total vector potential 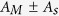 acting on *K* electrons is distinct in amplitude from its counterpart for 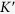 electrons^15^. That is to say, a valley gap comes into being [see the green ellipse dashed lines in Fig. 3(a)], where only electron state from one valley is propagating wave and that from the other valley is evanescent at a fixed Fermi energy. Thus the valley polarization can be achieved as long as the Fermi energy located inside the valley gap, akin to that in the strained graphene with a periodic magnetic modulation^15^. In addition, spin polarization is absent in such strained graphene system with magnetic barrier due to spin degeneracy. In Fig. 3(b). One can clearly see that the RSOC makes the spin degeneracy lifted and produces a spin energy gap. As a result, the spin polarization for single valley can be achieved in the spin energy gap^9^,^10^. But such system is not able to generate a valley-polarized current because of the mirror symmetry of *K* and 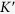 valleys^10^.

So we study the band structure of strained graphene with both magnetic barrier and RSOC [Fig. 3(c)]. It is shown that valley gap is induced in the energy range 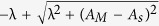 to 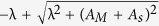 [see the green ellipse dashed lines in Fig. 3(c)]. Moreover, we also find that only single spin band at 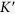 valley contributions to the current in that energy range as shown in Fig. 3(c), which leads to only one spin-flipped conductance for one valley is dominate [Fig. 2(a,b)]. Thus, high valley- and spin-polarization currents can be expected at those energies [Fig. 2(c)]. In addition, because the above band structure only for 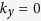, while the overall conductance is contributed from electrons with not only 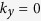, but also 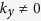, which lead the current can flow, even when the Fermi energy is located in the band gap near the 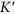 points [as seen in Fig. 2(a,c)]. The band structure also shows that the valley and single spin band gaps disappear for high Fermi energy, which lead to the valley and spin polarizations become lower and eventually converge to zero with increasing the Fermi energy. As a result, to observe valley/spin polarization current in the concerned structure, it is highly desirable to keep the system with valley gap and single spin band gap.

**Figure 4:**
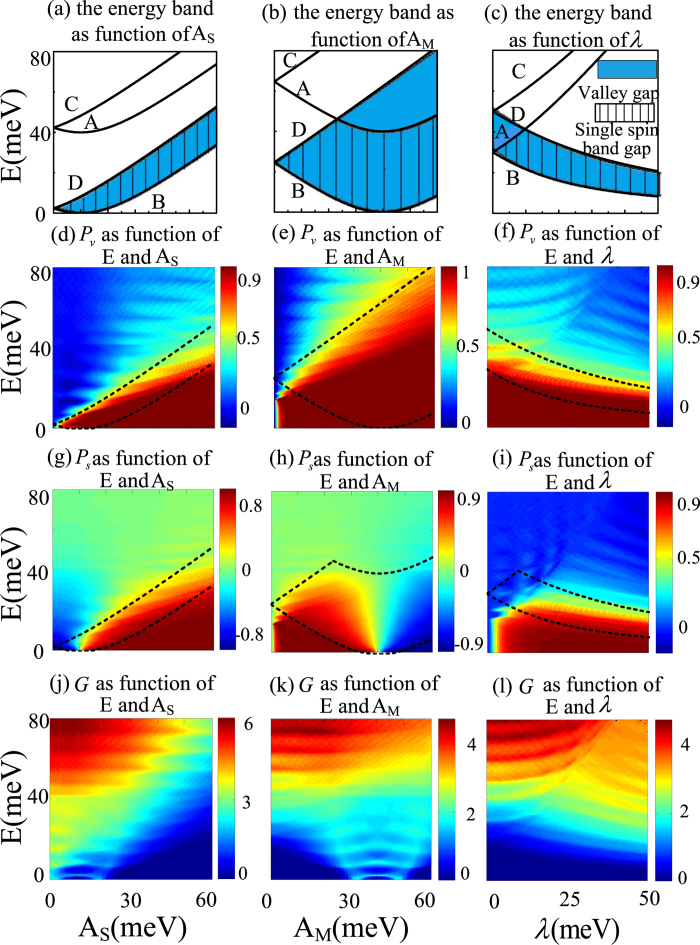
(**a**–**c**) The extreme points (A, B, C and D) of energy band as functions of strain, magnetic and RSOC strengths, (**d**–**f**) valley polarization, (**g**–**i**) spin polarization and (**j**–**l**) total conductance as functions of the Fermi energy *E* and strain, magnetic and RSOC strengths. The other parameters are 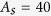 meV, 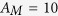 meV, 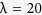 meV, 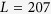 nm.

The valley and single spin band gaps can be deceived by the extreme points of energy band (A, B, C and D points) [see in Fig. 3(c)]. In Fig. 4(a–c), we plot the extreme points of energy band as functions of strain, magnetic barrier and RSOC strengths. With increasing the strain and magnetic strengths, we find that the valley gap firstly widens and then has no obvious change, and the location of the valley gap is shifted to high-energy region [Fig. 4(a,b)]. While the increase of RSOC strength makes the valley gap narrow and the location of the valley gap move toward low-energy region gradually [Fig. 4(c)]. In addition, it is shown that the single spin band gap coincides with valley gap for all Fermi energy with the increasing of the strain strength [Fig. 4(a)]. With increasing the magnetic strength, the single spin band gap firstly coincides with valley gap and then is narrower than the valley gap [Fig. 4(b)]. And the increase of RSOC strength causes that the single spin band gap firstly is smaller than valley gap and then coincides with valley gap [Fig. 4(c)]. The gap characters reflect on the valley/spin polarization [Fig. 4(d–i)]. In addition to the high valley/spin polarization in transmission gap, we can also find that the high valley polarization is mainly appears in the valley gap [Fig. 4(d–f)], and the high spin polarization mostly occur in the single spin band gap [Fig. 4(g–i)]. However, effective valley/spin filtering also requires a high transmitted valley/spin current. From Fig. 4(j–l), one can find in the transmission gap region, the total conductance is small. While within the valley gap or single spin band gap region, the total conductance can be larger than 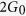, allowing for a remarkable valley/spin current, which confirming that such a strained graphene with valley gap and single spin band gap is an effective valley/spin filtering device. Moreover, more abundant spin polarization features of the system can be find in Fig. 4(g,h), where both high positive and negative spin polarizations are present with increasing the strain and magnetic strengths. Then, one can find that the negative spin polarization always occurs if 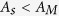, and the positive one appearing, provided 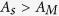. The reason can be understood as follows. Firstly, from Fig. 2, we can find that the presence of spin polarization is mainly caused by spin-flipped transmissions from 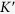 valley. The Hamiltonian of strain combined with magnetic vector for 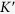 valley is 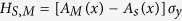. It is obvious that shifts of the Dirac points have the opposite direction for 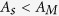 and 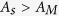. This feature leads to the transmission of electrons shows a mirror symmetry in 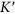 valley which causes the positive spin polarization is present for 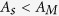 and negative spin polarization appears at 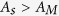. The above features show that the spin filtering properties can easily be switched by adjusting the strain and magnetization strengths.

## Summary

In conclusion, we have demonstrated that strained graphene with RSOC and magnetic barrier can generate valley- and spin-polarization currents. To observe such polarized current in the concerned structure, it is highly desirable to keep the system with valley gap and single spin band gap. The full valley-polarization current mainly appears in the valley gap and full spin-polarization one mostly occurs in the single spin band gap. The valley and single spin band gaps can be modulated by strain, RSOC and magnetic strength. In addition, under the strain and magnetic manipulation, the spin polarization can be switched by adjusting the strain and magnetization strengths. The full valley- and spin-polarization currents provide the desirable routines to construct spin/valley filter for graphene-based logic applications.

Our results would be also applicable to other spin and valley coupled systems, such as monolayers of silicene and MoS_2_, where low energy physics are governed by massive Dirac fermion.

## Additional Information

**How to cite this article**: Wu, Q.-P. *et al*. Full Valley and Spin Polarizations in Strained Graphene with Rashba Spin Orbit Coupling and Magnetic Barrier. *Sci. Rep.* **6**, 21590; doi: 10.1038/srep21590 (2016).
