# Corrigendum: Full Valley and Spin Polarizations in Strained Graphene with Rashba Spin Orbit Coupling and Magnetic Barrier

**DOI:** 10.1038/srep41199

**Published:** 2017-01-23

**Authors:** Qing-Ping Wu, Zheng-Fang Liu, Ai-Xi Chen, Xian-Bo Xiao, Zhi-Min Liu

Scientific Reports 6: Article number: 2159010.1038/srep21590; published online: 02 22 2016; updated: 01 23 2017

This Article contains errors in Reference 15 which was incorrectly given as:

Song, Y., Zhai, F. & Guo, Y. Strain-tunable spin transport in ferromagnetic graphene junctions. *Appl. Phys. Lett*. **103**, 183111 (2013).

The correct reference is listed below:

Song, Y., Zhai, F. & Guo, Y. Generation of a fully valley-polarized current in bulk graphene. *Appl. Phys. Lett*. **103**, 183111 (2013).

